# Ship Detection in SAR Image Based on the Alpha-stable Distribution

**DOI:** 10.3390/s8084948

**Published:** 2008-08-22

**Authors:** Changcheng Wang, Mingsheng Liao, Xiaofeng Li

**Affiliations:** 1 State Key Laboratory of Information Engineering in Survey, Mapping and Remote Sensing (LIESMARS), Wuhan University, Wuhan, Hubei 430079, P. R. China; 2 NOAA Science Center, WWBG, Room 102, 5200 Auth Road, Camp Springs, MD 20746, U.S.A. E-Mails: wchch1010@gmail.com (Changcheng Wang); liao@whu.edu.cn (Mingsheng Liao); Xiaofeng.Li@noaa.gov (Xiaofeng Li)

**Keywords:** Alpha-stable distribution, ship detection, Synthetic Aperture Radar, Constant False Alarm Rate (CFAR)

## Abstract

This paper describes an improved Constant False Alarm Rate (CFAR) ship detection algorithm in spaceborne synthetic aperture radar (SAR) image based on Alpha-stable distribution model. Typically, the CFAR algorithm uses the Gaussian distribution model to describe statistical characteristics of a SAR image background clutter. However, the Gaussian distribution is only valid for multilook SAR images when several radar looks are averaged. As sea clutter in SAR images shows spiky or heavy-tailed characteristics, the Gaussian distribution often fails to describe background sea clutter. In this study, we replace the Gaussian distribution with the Alpha-stable distribution, which is widely used in impulsive or spiky signal processing, to describe the background sea clutter in SAR images. In our proposed algorithm, an initial step for detecting possible ship targets is employed. Then, similar to the typical two-parameter CFAR algorithm, a local process is applied to the pixel identified as possible target. A RADARSAT-1 image is used to validate this Alpha-stable distribution based algorithm. Meanwhile, known ship location data during the time of RADARSAT-1 SAR image acquisition is used to validate ship detection results. Validation results show improvements of the new CFAR algorithm based on the Alpha-stable distribution over the CFAR algorithm based on the Gaussian distribution.

## 1. Introduction

Synthetic Aperture Radar (SAR) is an active radar that can provide high resolution images in the microwave band under all weather conditions. SAR images have been widely used for fishing vessel detection, ship traffic monitoring and immigration control [1,2]. Numerous studies have been performed to develop ship detection algorithms in SAR images automatically. Ships can be identified as hard targets or by their wakes [3,4,5] in the SAR image. In general, the Radar Cross Section (RCS) of ships is higher than the surrounding sea clutter. This is due to the effect of multiple bounces of incoming radar waves from the ships superstructure [6]. Therefore, ships can be separated from the sea cluster with an appropriate choice of RCS threshold. The Constant False Alarm Rate (CFAR) algorithm is widely used for computing the adaptive threshold [5,7]. To use the CFAR algorithm, one must first determine an appropriate probability density function (PDF) that can adequately describe the statistical characteristics of background clutter. For multilook SAR images, a commonly used PDF is the Gaussian distribution. As demonstrated in reference [8], for a multilook SAR intensity image, when the number of looks is over 30, it is suitable to assume the Gaussian PDF of clutter in the multilook image. Therefore, The Gaussian distribution is only valid when a large number of radar looks are averaged [1]. Other distributions such as Gamma or K- distribution have also been suggested [9, 10], but they carry the same limitations as the Gaussian distribution. In general, as sea clutter in SAR images always shows spiky or heavy-tailed characteristics, these distributions often fail to describe heavy-tailed sea clutter in many actual applications [11]. When using these distributions for modeling the sea clutter in a CFAR algorithm, we find that they either produce a lot of false alarms or miss some ship target detections.

The Alpha-stable distribution is a statistical model that is particularly applicable for radar returns from targets and sea clutter that are impulsive or spiky in nature [11]. In this paper, we use the Alpha-stable distribution to model the sea clutter in SAR images. An improved algorithm based on the Alpha-stable distribution is proposed for ship detection in SAR images. An image of RADARSAT-1 ScanSAR image and in situ ship position data are used to validate the proposed algorithm.

## 2. Methodology

### 2.1. CFAR Based Ship Detection Algorithm

The CFAR algorithm is widely used for setting a threshold so that we can find targets that are statistically significant above the background signal while maintaining a constant false alarm rate [1, 12]. A distribution function that fits distribution of region of interest of clutter is first computed for determining the threshold. Then all pixels with their values higher than the threshold are defined as ship targets. Let *f* (*x*) be the PDF of the background clutter. The probability of false alarm *P_fa_* and threshold T is given by the equation [5]:

(1) $$P_{fa}=\int_{T}^{\infty }f\;(x)dx=1-\int_{0}^{T}\;f(x)dx$$

For a specified probability of false alarm *P_fa_* and a PDF *f* (*x*) of background sea clutter, we can get the threshold *T* by solving Equation (1).

### 2.2. The Alpha-stable Distribution

The Alpha-stable distribution is widely used in the processing of impulsive or spiky signals [6,11,12,14,15]. The distribution is derived from the generalized central limit theorem (GCLT). In fact, the Gaussian distribution is a special case of the Alpha-stable distribution [14].

Due to the lack of closed-form formulas for PDFs, the Alpha-stable distribution is often described by its characteristic function, *φ(t)*, which is the Fourier transform of the PDF [14]:

(2) $$\varphi \left(t\right)=\{\begin{array}{ll} \text{exp}\;\left\{j\mu t-\gamma {\left|t\right|}^{\alpha }\left[1-j\beta \mathrm{sgn}(t)\tan\frac{\pi \alpha }{2}\right]\right\} & \text{if}\;\alpha \neq 1\\ \exp\;\left\{j\mu t-\gamma \left|t\right|\left[1+j\frac{2}{\pi }\beta \mathrm{sgn}(t)\log\left|t\right|\right]\right\} & \text{if}\;\alpha =1 \end{array}$$

*φ(t)* depends on four parameters: the characteristic exponent, αЄ(0,2), measuring the thickness of the tails of the distribution (the smaller the values of a, the thicker the tails of distribution are); the symmetry parameter, βЄ[-1,1] setting the skewness of the distribution; the scale parameter, γ>0; and the location parameter, μЄR. In general, no closed-form expression exists for the Alpha-stable distribution, except for the Gaussian (α=2), Cauchy (α=1, β=0), and Pearson (α=0.5, β=-1) distributions. When β=0 and μ=0, the distribution is called a symmetric Alpha-stable (*S*α*S*) distribution. Its characteristic function is then simplified as [14]:

(3) $$\varphi \left(t\right)=\exp\left(-\gamma {\left|t\right|}^{\alpha }\right)$$

The four parameters in the Alpha-stable distribution can not be directly derived from conventional statistical methods, Instead, several numerical methods, such as the maximum likelihood, sample fractiles or negative-order moments, have been developed to solve these parameters [14]. In this study, we apply a regression-type numerical method developed by Koutrouvelis [16] to estimate these four parameters α, γ, β and μ.

To calculate the PDF *f* (*x;α,β,γ,μ*) of Alpha-stable distribution, we can firstly compute the PDF *f* (*z;α,β*) of the standard Alpha-stable distribution with two parameters α and β by using the formula*z* = (*x*−*μ*)/*γ*^1/α^. By taking the inverse Fourier transform of the characteristic function, the PDF of the standard Alpha-stable distribution is given by [14]

(4) $$f\left(z;\alpha ,\beta \right)=\{\begin{array}{cc} \frac{1}{\pi }\int_{0}^{\infty }\exp\left(-t^{\alpha }\right)\cos\left[zt+\beta t^{\alpha }\;\tan\left(\frac{\pi \alpha }{2}\right)\right]dt & \mathrm{if}\;\alpha \neq 1\\ \frac{1}{\pi }\int_{0}^{\infty }\exp\left(-t\right)\cos\left[zt+\frac{2}{\pi }\beta t\;\log(\left|t\right|)\right]dt & \mathrm{if}\;\alpha =1 \end{array}$$

Nolan [17] proposed a numerical integral method for calculating the PDF of *f* (*x;α,β,γ,μ*). We apply it to calculate the PDF of Alpha-stable distribution.

### 2.3. CFAR Algorithm Based on the Alpha-stable Distribution

**Figure 1.**
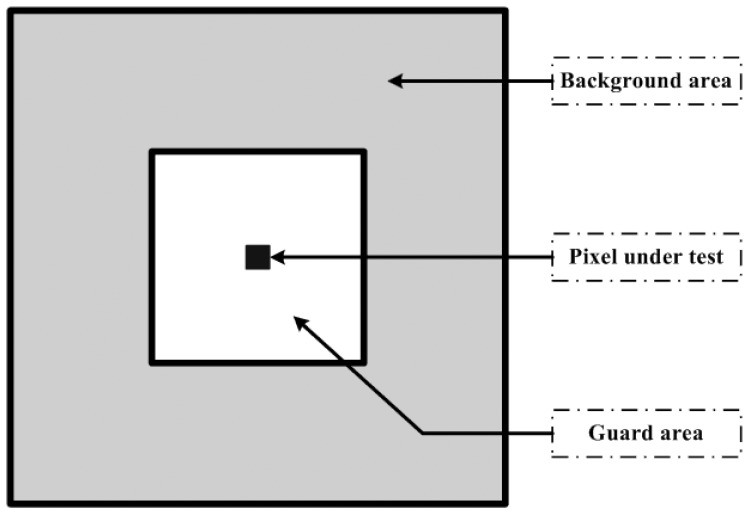
Sliding windows for CFAR algorithm.

**Figure 2.**
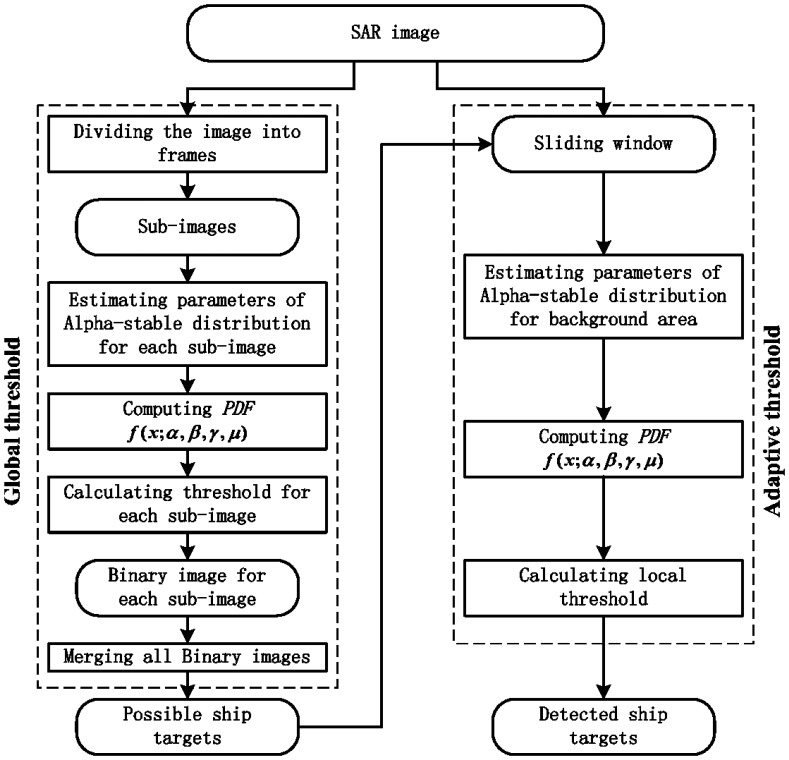
Flowchart of the proposed algorithm.

The two-parameter CFAR algorithm is widely used for ship detection [18]. The core of this algorithm is a local process to search for bright pixels that are statistically different from surrounding sea clutter. This is done by calculating an adaptive threshold according to the statistics of the surrounding sea clutter. The surrounding area is always set as a “ring” (the gray area in Figure 1) around the pixel being tested. This “ring” is moved one pixel each time across the whole image, which is time consuming. To alleviate this problem, an initial step for detecting possible ship targets is employed in our proposed algorithm. Then, a local process is applied to the pixel identified as a possible target by the initial step. Figure 2 shows the flowchart of our proposed algorithm.

In the first step, the SAR image is divided into a number of frames of equal size and each frame is processed as a whole. Sea clutter in each frame is assumed to be relatively homogeneous [2]. The image samples in the whole frame are used to estimate the four parameters (α, γ, β and μ) of the Alpha-stable distribution using a regression-type method developed by Koutrouvelis [16]. Then the PDF *f* (*x;α,β,γ,μ*) of the Alpha-stable distribution is computed by a numerical integral method and the threshold is calculated by (1). This threshold is then applied to the whole frame. Pixels with values above this threshold are considered as possible targets and labeled as ‘1’ in a binary image. The identified targets from all frames in the SAR image are combined as the initial ship detection result. It is worth noting that the constant false alarm rate *P_fa_* in this initial step needs to be set higher than the commonly used value (such as 0.001‰), which ensures that all possible targets will be detected in this step.

In the second step, a local process is applied to the pixels labeled as ‘1’ after the first step. Similar to the two-parameter CFAR algorithm, we define two windows: a guard window nested within a background window. Both windows are centered at the pixel being tested. The background “ring” (the gray area in Figure 1) between the guard and background windows contains sea clutter samples. The purpose of the guard window is to ensure that no pixel of an extended ship target is included in the background “ring” [5]. Therefore, the background “ring” is purely representative of background sea clutter around the pixel being tested. We run the first step again on the data within the “ring” to find the threshold. If the value of the pixel tested is higher than this threshold, it is a verified ship target.

## 3. Experimental Results

### 3.1. Study Areas and Data

**Figure 3.**
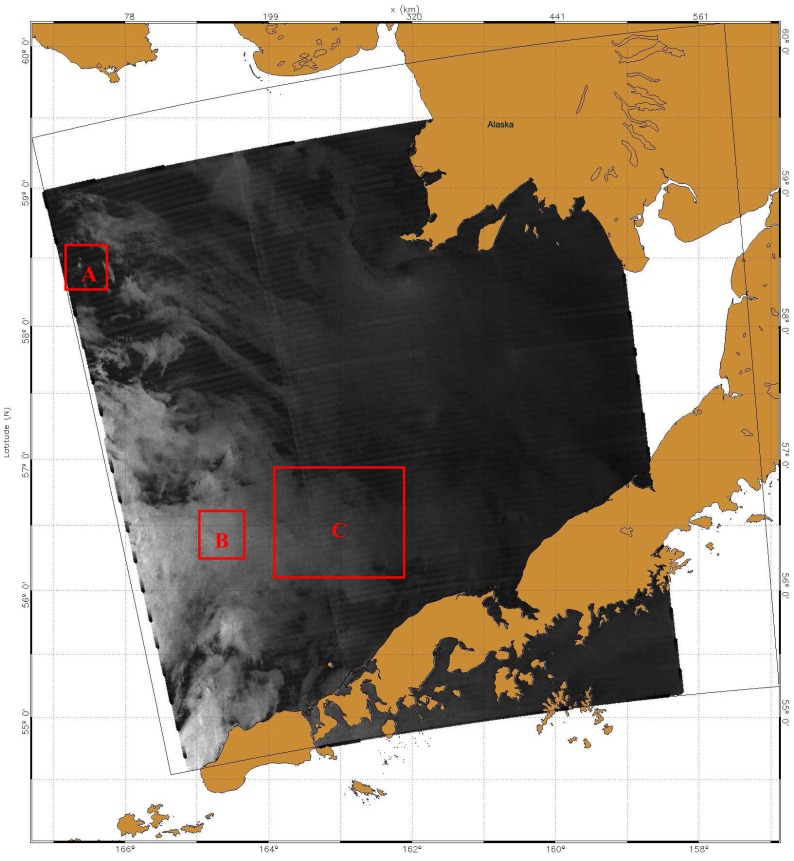
RADARSAT-1 ScanSAR image (© CSA 2002). Three rectangle boxes indicate study areas A, B and C.

In October 1999, National Oceanic and Atmospheric Administration (NOAA), USA, initialized a program called the Alaska SAR Demonstration (AKDEMO) [1]. One of the important AKDEMO goals is to provide ship detection products for users in Alaska in near real time. The RADARSAT-1 SAR image used in this study was obtained from the AKDEMO program. Figure 3 is an image of RADARSAT-1 ScanSAR Wide-B Mode image with a 450 kilometers swath width. The image was acquired at 04:28:52 GMT on October 16. 2002. Its spatial resolution is 100 m and the pixel spacing is 50 m. The image shows coastal lands of Alaska and Bering Sea. Three red rectangle boxes **A** (437 by 470 pixels), **B** (350 by 350 pixels) and **C** (1850 by 2100 pixels) indicate three study areas.

### 3.2. Test for Alpha-stable Distribution

**Figure 4.**
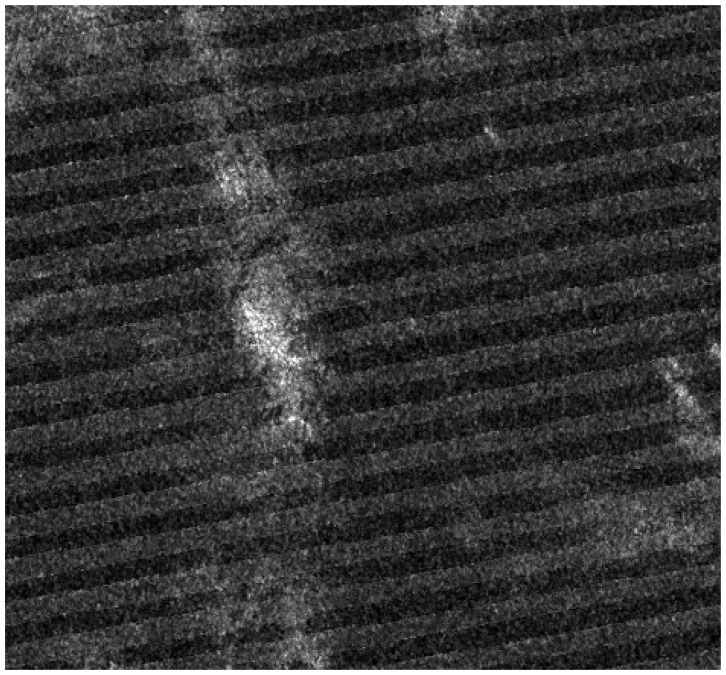
RADARSAT-1 sub-image of the study area **A** (© CSA 2002).

Figure 4 shows the RCS image of study area **A**. It shows a spiky sea clutter area that is used for comparing different distribution models. The estimated parameters of Gaussian, K- and Alpha-stable distributions for this area are listed in Table 1. For the Gaussian distribution, parameters μ and σ represent mean and standard deviation. For the K-distribution, *L* is the number of statistically independent looks, and *ν* is a shape parameter [13].

**Figure 5.**
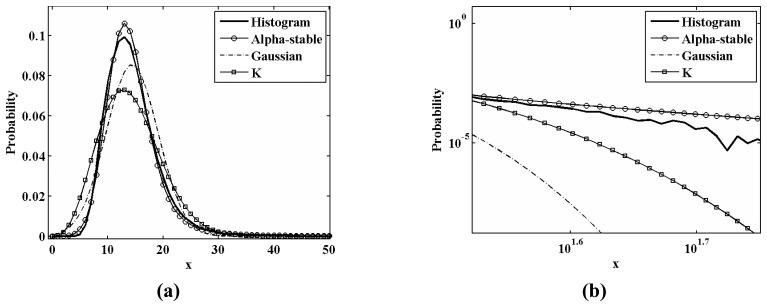
The histogram and PDFs of various distributions for the study area **A: (a)** the over all curves on a linear scale; **(b)** the tails of the curves on a log-log scale.

Figure 5(a) shows the histogram and PDFs of various distributions on a linear scale for the study area **A**. Figure 5(b) shows the tails of the curves on a log-log scale. The dashed line represents the PDF of the Gaussian distribution. The dashed line with open squares represents the PDF of K-distribution. The line with open circles represents the PDF of the Alpha-stable distribution. One can see that the PDF of the Alpha-stable distribution fits the histogram much closer than those of the Gaussian and K-distributions. In addition, it is worth noting that the PDF of the Alpha-stable distribution has heavier tail than these of the other two distributions.

**Figure 6.**
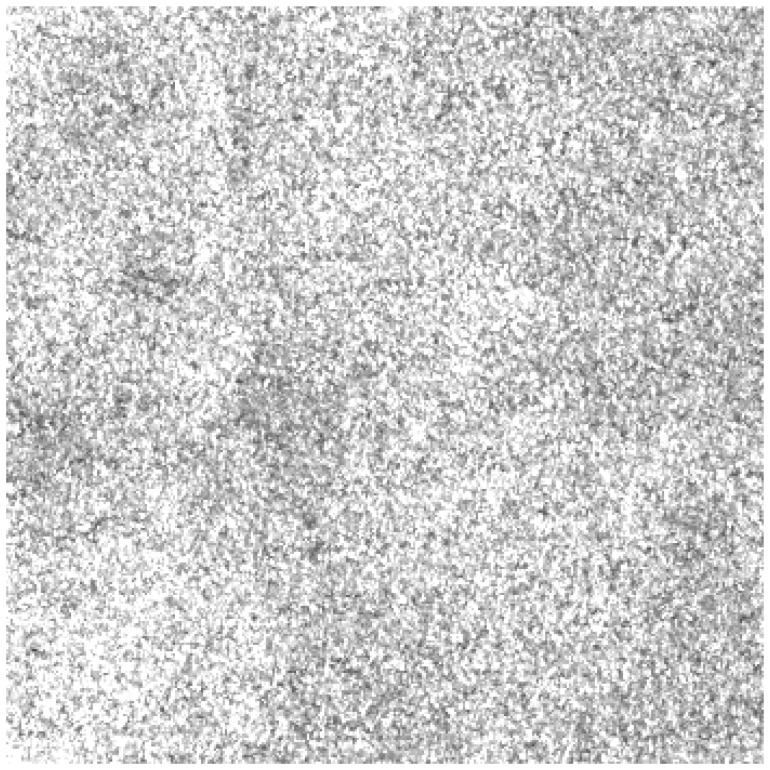
RADARSAT-1 sub-image of study area B (© CSA 2002).

**Figure 7.**
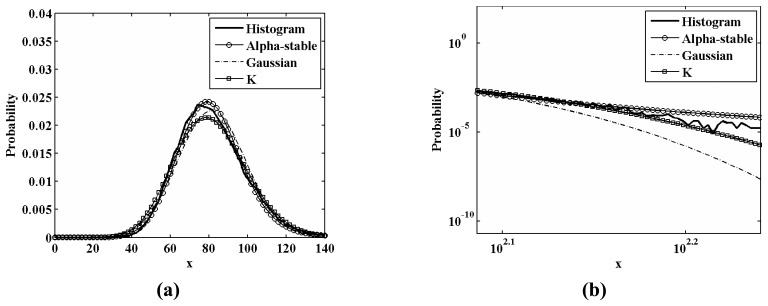
The histogram and PDFs of various distributions for study area **B: (a)** the over all curves on a linear scale; **(b)** the tails of the curves on a log-log scale.

Figure 6 also shows a sea clutter area (study area **B**) with a mean RCS value of much higher than that of the study area **A**. The estimated parameters of three distributions for this area are also listed in Table 1. Figure 7 shows the histogram and PDFs of various distributions for the study area **B**. One can also see that the PDF of the Alpha-stable distribution has a heavier tail than that of the other two distributions. Therefore, we conclude that the Alpha-stable distribution fits the statistics of sea clutter better than the other two distributions.

### 3.3. Validation of Ship Detection Results

**Figure 8.**
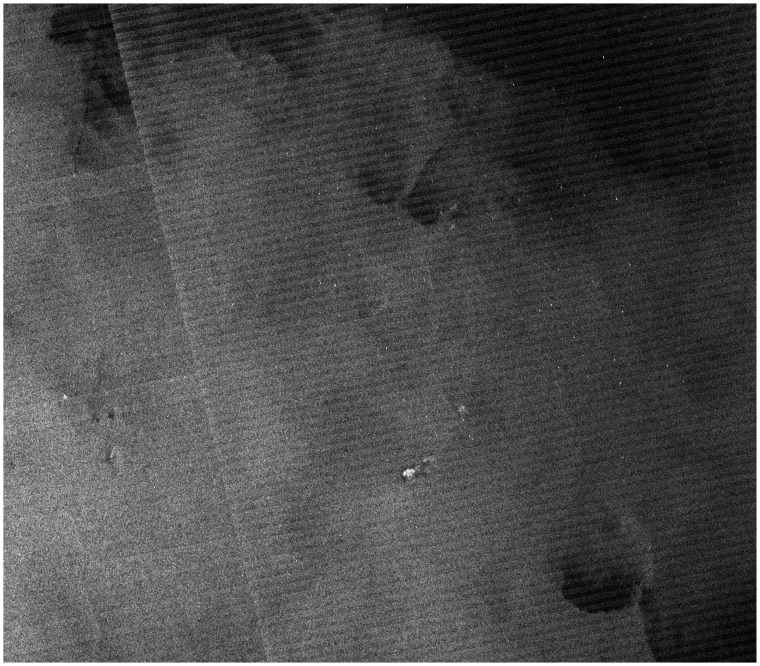
RADARSAT-1 sub-image (© CSA 2002) of the study area **C**.

Figure 8 shows the RCS image of study area **C**. The image covers an area of about 100 km by 90 km. In the study area **C**, there were 13 ship locations recorded by observers in these ships during the time of the RADARSAT-1 image acquisition. Table 2 lists information about these ships. The length of these ships ranges from 26.8 to 50.6 m. This information is used to validate the ship detection algorithm.

**Figure 9.**
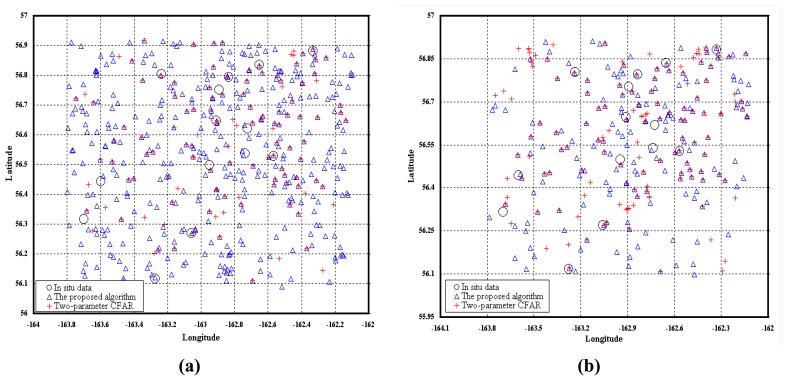
Map of detected ships produced by the proposed algorithm and Two-parameter CFAR algorithm with different sets of windows. **(a)** The signal, guard and background windows are set as 5 by 5, 9 by 9 and 25 by 25, respectively. **(b)** The signal, guard and background windows are set as 5 by 5, 13 by 13 and 41 by 41, respectively.

Figure 9 shows *in situ* ship locations recorded by observers in the ships (black circles), the results of a two-parameter CFAR algorithm (red pluses) and the results of our proposed algorithm (blue triangles) with different sets of windows. In our proposed algorithm, the frame size in the first step is set as 100 by 100. In Figure 9(a), the guard and background windows are set as 9 by 9 and 25 by 25, respectively. The value of CFAR *P_fa_* in the first step is set as 1‰. This value is relatively higher than that of commonly used (such as 0.001‰). The value of constant false alarm rate *P_fa_* in the second step is set as 0.001‰. In the two-parameter CFAR algorithm (based on the Gaussian distribution), the signal, guard and background windows are set as 5 by 5, 9 by 9 and 25 by 25, respectively [1]. The threshold ***T****_o_* is set to 2.0 which is a relatively lower value than that mentioned in reference [1].

Due to the time differences between the RADARSAT-1 image and validation observations, geocoding errors in the RADARSAT-1 image and possible differences in the reference datum [2], there usually exists some deviation between the locations of the detected ships and the reported locations. As a result, a particular ship is not considered identified if its location is more than 3 km away from the *in situ* reported location [2]. The ship validation information for our proposed algorithm and the two-parameter CFAR algorithm for the study area **C** is listed in Table 3. The column “Distance (km)” in Table 3 represents the distance between a certain reported ship location and its nearest detected ship target. 12 out of 13 validation ships are detected by our proposed algorithm and 9 out of 13 are detected by the two-parameter CFAR algorithm. However, our proposed algorithm produced more false alarms than the two-parameter CFAR algorithm did. The main reason is that a relatively large number (thousands) of samples are necessary to accurately estimate parameters of Alpha-stable distribution. Thus we set the guard and background windows as 13 by 13 and 41 by 41, respectively. The corresponding results of the two algorithms are shown in Figure 9(b); also, the detailed ship validation information is listed in Table 3. 12 out of 13 validation ships are detected by our proposed algorithm and 11 out of 13 are detected by the two-parameter CFAR algorithm. Since only partial ground truth information is available for the study area **C**, it is difficult to compute the real probability of detection and false alarm. Therefore, the probability of a false alarm is estimated by counting the number of false alarms through visual analysis, and then divided by the number of possible placements of the background window in the subset [1]. In Figure 9(b), for the result of our proposed algorithm, the estimated number of false alarms and detected ships is 52 and 149, respectively; for the two-parameter CFAR algorithm, that is 53 and 92, respectively. Then the estimated false alarm probabilities of the two algorithms are 0.0134‰ and 0.0136‰, respectively. Though the estimated false alarm probabilities are almost the same, the number of the ships detected by our proposed algorithm is larger than that of the two-parameter CFAR algorithm.

## 4. Conclusions

An improved CFAR ship detection algorithm based on the Alpha-stable distribution is proposed in this paper. First, we demonstrated the advantage of using the Alpha-stable distribution over Gaussian and K- distributions for ship detection. The results show that the Alpha-stable distribution was capable of modeling a spiky distribution with heavy (thick) tails, such as the distribution of the sea clutters in a SAR image. Then, a RADARSAT-1 ScanSAR Wide-B Mode image and in situ ship position data are used to validate the proposed algorithm. Compared with typical two-parameter CFAR algorithm (which is based on Gaussian distribution), the proposed algorithm showed an improvement in detecting ships in the high spiky sea clutter background SAR image.

## Figures and Tables

**Table 1.** The estimated parameters of different distributions from sub-area in A and B respectively.

Study Area	Distributions	Parameter 1	Parameter 2	Parameter 3	Parameter 4
**A**	Gaussian	μ= 14.3882	σ= 4.6616	-	-
	K	L= 1.8140	ν= 98.4250	-	-
	Alpha-stable	α= 1.8067	γ= 6.3132	β= 1.0000	μ= 14.7815
**B**	Gaussian	μ= 81.3233	σ= 17.6446	-	-
	K	L= 5.0781	ν= 98.4250	-	-
	Alpha-stable	α= 1.9560	γ= 129.9372	β = 1.0000	μ= 81.8972

**Table 2.** Ship locations at the time of the RADARSAT-1 SAR image.

No.	Ship name	Date (GMT)	Time (GMT)	Latitude	Longitude	Ship length (m)	Wind speed (m/s)	Sea state
1	Gladiator	2002-10-16	4:28:39	56.117	-163.275	37.8	5.14	4
2	Alliance	2002-10-16	4:28:39	56.270	-163.058	30.5	5.14	2
3	Andronica	2002-10-16	4:28:39	56.317	-163.698	30.2	-	3
4	Alaska Sea	2002-10-16	4:28:39	56.443	-163.598	33.5	-	calm
5	Pavlof	2002-10-16	4:28:39	56.497	-162.948	50.6	-	1
6	Handler	2002-10-16	4:28:39	56.528	-162.568	38.4	1.03	calm
7	Sultan	2002-10-16	4:28:39	56.538	-162.737	39.6	5.14	<3
8	Argosy	2002-10-16	4:28:39	56.646	-162.910	37.8	5.14	calm
9	Kelveen K	2002-10-16	4:28:39	56.751	-162.892	32.0	7.72	6
10	Northwind	2002-10-16	4:28:39	56.794	-162.836	32.0	2.57	<4
11	Early Dawn	2002-10-16	4:28:39	56.803	-163.239	32.9	2.24	3
12	Aleutian Beauty	2002-10-16	4:28:39	56.839	-162.652	29.9	2.57	1
13	Big Blue	2002-10-16	4:28:39	56.882	-162.330	26.8	0	<4

**Table 3.** The results of validated ships detection for the study area **C**. (BW: Background Window; GW: Guard Window; SW: Signal Window).

No.	Ship name	The proposed algorithm (BW: 25×25, GW:9×9)		Two-parameter CFAR (BW:25×25, GW:9×9, SW:5×5)		The proposed algorithm (BW:41×41, GW:13×13)		Two-parameter CFAR (BW:41×41, GW:13×13, SW:5×5)	
		Detection	Distance (km)	Detection	Distance (km)	Detection	Distance (km)	Detection	Distance (km)
1	Gladiator	Yes	0.356	**No**	**9.351**	Yes	0.356	Yes	0.356
2	Alliance	Yes	0.255	Yes	0.255	Yes	0.255	Yes	0.255
3	Andronica	**No**	**3.211**	**No**	**3.211**	**No**	**3.211**	**No**	**3.211**
4	Alaska Sea	Yes	0.786	**No**	**4.710**	Yes	0.786	Yes	1.555
5	Pavlof	Yes	1.740	Yes	1.740	Yes	1.740	Yes	1.740
6	Handler	Yes	0.186	Yes	0.186	Yes	0.186	Yes	0.186
7	Sultan	Yes	0.867	**No**	**5.534**	Yes	0.867	**No**	**5.563**
8	Argosy	Yes	0.914	Yes	0.914	Yes	0.914	Yes	0.914
9	Kelveen K	Yes	2.201	Yes	2.201	Yes	2.201	Yes	2.201
10	Northwind	Yes	0.261	Yes	0.261	Yes	0.261	Yes	0.261
11	Early Dawn	Yes	0.375	Yes	0.375	Yes	0.375	Yes	0.375
12	Aleutian Beauty	Yes	0.237	Yes	0.237	Yes	0.237	Yes	0.237
13	Big Blue	Yes	0.629	Yes	0.629	Yes	0.629	Yes	0.629
